# Intravenous Vitamin C as an Add-on Therapy for the Treatment of Sepsis in an Intensive Care Unit: A Prospective Cohort Study

**DOI:** 10.3390/medicina60030464

**Published:** 2024-03-12

**Authors:** Sergio Antonio Gonzalez-Vazquez, Eli Efrain Gomez-Ramirez, Laura Gonzalez-Lopez, Jorge Ivan Gamez-Nava, Juan Angel Peraza-Zaldivar, Aline Priscilla Santiago-Garcia, Melissa Ramirez-Villafaña, Fabiola Gonzalez-Ponce, Jose Jorge Gomez-Camarena, Ana Miriam Saldaña-Cruz, Norma Alejandra Rodriguez-Jimenez, J. Ahuixotl Gutierrez-Aceves, Adriana Jimenez-Lopez, Sylvia Elena Totsuka-Sutto, Ernesto German Cardona-Muñoz, Juan Manuel Ponce-Guarneros

**Affiliations:** 1 Programa de Doctorado en Farmacología, Centro Universitario de Ciencias de la Salud, Universidad de Guadalajara, Guadalajara 44340, Mexico; sergiogonvaz@yahoo.com.mx (S.A.G.-V.); p_juan_angel@hotmail.com (J.A.P.-Z.); aline.santiago@investigacionmedica.com.mx (A.P.S.-G.); jorgegomez59@gmail.com (J.J.G.-C.); ahuixotl@gmail.com (J.A.G.-A.); 2 Unidad de Cuidados Intensivos, Hospital General Regional 110, Instituto Mexicano del Seguro Social, Guadalajara 44716, Mexico; 3 Instituto de Terapéutica Experimental y Clínica, Departamento de Fisiología, Centro Universitario de Ciencias de la Salud, Universidad de Guadalajara, Guadalajara 44340, Mexico; dr.efrain.gomez@gmail.com (E.E.G.-R.); lauraacademicoudg@gmail.com (L.G.-L.); ivangamezacademicoudg@gmail.com (J.I.G.-N.); melissa.ramirez@academicos.udg.mx (M.R.-V.); fgponce.ln@gmail.com (F.G.-P.); ana.saldanac@academicos.udg.mx (A.M.S.-C.); norma.rodriguezj@academicos.udg.mx (N.A.R.-J.); stotsuka@hotmail.com (S.E.T.-S.); cameg1@gmail.com (E.G.C.-M.); 4 Programa de Maestría en Salud Publica, Centro Universitario de Ciencias de la Salud, Universidad de Guadalajara, Guadalajara 44340, Mexico; 5 Especialistas en Medicina Critica, Hospital Terranova, Guadalajara 44670, Mexico; adrianajl1878@gmail.com

**Keywords:** sepsis, vitamin C, mortality

## Abstract

*Background and Objectives*: According to the Third International Consensus Definitions for Sepsis and Septic Shock (Sepsis-3), sepsis is defined as “life-threatening organ dysfunction caused by a dysregulated host response to infection”. The increased presence of free radicals causes an increase in oxidative stress. Vitamin C is an essential water-soluble vitamin with antioxidant activity and immunoregulatory effects that plays a potential role in the treatment of bacterial infections. Our aim was to evaluate the effectiveness of adding vitamin C to the conventional treatment of sepsis to decrease its mortality rate. *Materials and Methods:* In a prospective cohort study, we included patients with a diagnosis of sepsis and a SOFA score ≥ 9 who were evaluated in an Intensive Care Unit at a secondary-care hospital. According to the intensive care specialist, they were treated using two different strategies: Group 1—patients with sepsis treated with conventional treatment without vitamin C; Group 2—patients with sepsis with the addition of vitamin C to conventional treatment. *Results:* We included 34 patients with sepsis. The incidence of mortality was 38%, and 47% of patients used vitamin C as an adjuvant to the basic treatment of sepsis. In the basal analyses, patients treated with use of vitamin C compared to patients treated without vitamin C required less use of glucocorticoids (75% vs. 100%, *p* = 0.039). At follow-up, patients treated without vitamin C had higher mortality than patients treated with vitamin C as an adjuvant for the treatment of sepsis (55.6% vs. 18.8%, *p* = 0.03). We observed that the use of vitamin C was a protective factor for mortality in patients with sepsis (RR: 0.54, 95% CI: 0.31–0.96, *p* = 0.03). *Conclusions:* The use of vitamin C as an adjuvant to treatment decreases the risk of mortality by 46% in patients with sepsis and SOFA ≥ 9 compared to patients treated without vitamin C as an adjuvant to sepsis.

## 1. Introduction

According to the Third International Consensus Definitions for Sepsis and Septic Shock (Sepsis-3), sepsis is defined as “life-threatening organ dysfunction caused by a dysregulated host response to infection” [1]. Sepsis is estimated to cause approximately 5.3 million deaths per year worldwide [2].

For the evaluation of the severity of sepsis, the Sequential Organ Failure Assessment (SOFA) severity scale was implemented, which considers clinical characteristics, laboratory results, and treatment [1]. The systemic alterations that occur during sepsis are mainly caused by an increase in proinflammatory cytokines affecting the microvascular level, hemodynamic imbalance, and coagulation disorders, and ultimately causing organic dysfunction [3]. Oxidative stress increases with the increasing presence of free radicals, such as superoxide anion, hydrogen peroxide, and hydroxyl radicals (derived from the activation of LI-1, IL-6, and TNF-a), which block the cellular respiration chain, resulting in mitochondrial permeability and cell apoptosis [4].

Vitamin C (ascorbic acid) is an essential water-soluble vitamin that has antioxidant activity, since it can decrease the activity of NADPH oxidase (NOX); on the other hand, its regulatory effects on the immune system can regulate the response of macrophages by decreasing the production of proinflammatory factors and exerting bacteriostatic effects on pathogens [5].

The benefit of using intravenous (IV) vitamin C as an adjuvant in the management of sepsis is still unclear, with some studies suggesting that it is useful [6], while others indicate a lack of effectiveness. In a controlled clinical trial conducted by Lamontagne, no differences in mortality and persistent organ dysfunction between the vitamin C group and the placebo group were observed after adjusting for different confounders [7]. Recently, four meta-analyses have been carried out examining the effectiveness of IV vitamin C in sepsis [8,9,10,11]. In the first meta-analysis, performed by Patel et al., the authors concluded that the use of a high-dose of IV vitamin C was associated with decreasing the rate of mortality in 30%; however, a low-dose of IV vitamin C did not exert changes in that outcome [8]. In a second meta-analysis, Wen et al. identified that IV vitamin C produces a non-significant trend toward improving the short-term mortality and overall mortality in patients with sepsis; however, their results did not achieve statistical significance [9]. The last two meta-analyses, performed by Sato et al. [10] and Cai et al. [11], did not identify benefits of administrating a high-dose of IV vitamin C in the short-term mortality [10], in-hospital mortality rate [11], or intensive care unit (ICU) mortality rate [11]. Therefore, there is a wide variability in the results of these four meta-analyses, explained by differences in the inclusion criteria, doses of IV vitamin C, time to follow-up, and moreover, by differences in the severity of sepsis at the time of the inclusion across the studies. We hypothesized that the benefits of adding a high-dose of IV vitamin C can be found mainly in patients with a high SOFA score at the time of the study entry. Therefore, the aim of our study was to evaluate the effectiveness of adding IV vitamin C to the conventional treatment of sepsis in patients with a high SOFA score (≥9 points) to decrease the 30-days mortality rate.

## 2. Materials and Methods

We carried out a prospective cohort study that included patients with a diagnosis of sepsis who were evaluated in an ICU at a secondary-care hospital (IMSS 110) between January 2022 and December 2023. The inclusion criteria were as follows: >18 years of age, SOFA score ≥ 9, and informed consent. We excluded patients with pancreatitis, polytrauma, burns, and COVID-19 infection, as well as those who were post-surgery or had received cardiopulmonary resuscitation during their hospital stay. Sepsis was diagnosed according to the SEPSIS-3 criteria [1].

### 2.1. Study Groups

According to the indicated therapy by the intensive care specialist, patients were divided into two groups: Group 1—patients with sepsis treated with conventional treatment (antibiotics, fluids, and medications to maintain the blood pressure, oxygen, and supporting measurements) without IV vitamin C; Group 2—patients with sepsis treated with the addition of IV vitamin C to conventional treatment.

### 2.2. Use of Vitamin C

This was an observational study, in which IV vitamin C was indicated by the attending intensive care specialist as an adjuvant to the basic treatment for the management of sepsis upon admission to the intensive care unit. Vitamin C was administered as an ascorbic acid dose of 2 g every 8 h (AMP 1 g/10 mL Infalet^®^, Pisa, Italy); 2 g of vitamin C was diluted in 100 mL of dextrose at 5% and administered by IV over a period of 30 min. Vitamin C therapy was indicated at the time of admission to the ICU and during the time that the patients stayed in the intensive care unit.

### 2.3. Clinical Assessments and Follow-Up

The assessments were performed at baseline, 24 h, 48 h, and 72 h. We evaluated the SOFA scale to identify respiratory problems, platelet alterations, liver disease, altered alertness, and kidney injury. The laboratory test for the study included blood count and blood chemicals (creatinine, urea, glucose). Follow-up was given until discharge from the hospital, with the objective of evaluating the 30-days mortality rate (main objective).

### 2.4. Outcome Measurements

The main outcome measure was the 30-day mortality rate. Additionally, we identified the following secondary outcomes: days since stay in the ICU, days of using mechanical ventilation, retired mechanical ventilation at 5 days, days of using vasoactive amines, reduction in vasoactive amines at 5 days, decreases in SOFA at 72 h, and mean arterial pressure (MAP) at 72 h.

### 2.5. Statistical Analysis

Quantitative variables are expressed as means and standard deviations, and qualitative variables as frequencies and percentages. Differences between groups were assessed using Chi^2^ and *t*-Student. We calculated relative risk (RR) for the analysis of intervening variables for mortality and the confidence interval (CI95%). Statistical significance was considered at the *p* ≤ 0.05 level.

### 2.6. Ethics

Our project was carried out following the terms of the Helsinki Declaration. A signed informed consent was requested from each patient or a family member on a voluntary basis. This project was approved by the Ethics and Research Committee of the Hospital (code of approval: R-2019-1305-130).

## 3. Results

**Figure 1:**
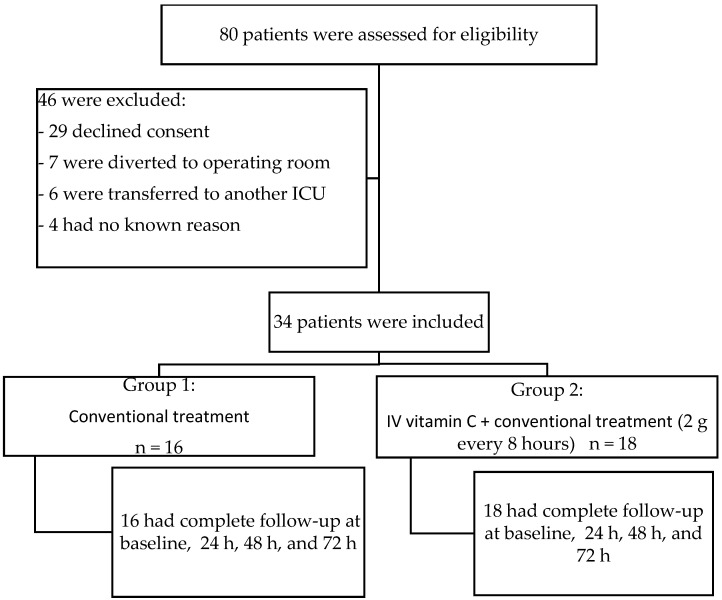
Flowchart of patient enrollment.

We identified 80 patients with a diagnosis of sepsis who were admitted to the ICU. Of these, 29 patients did not agree to participate, 7 were excluded because they were post-surgical, 6 were due to be transferred to another unit, and 4 were excluded for other reasons (Figure 1).

We included 34 patients with sepsis with a SOFA score at baseline ≥ 9. The causes of sepsis were pneumonia (60%), undetermined (20%), urinary tract infection (11.1%), endocarditis (2.2%), and sepsis associated with intravenous devices (2.2%). Only 4% of our patients experienced septic shock.

Table 1 shows the clinical characteristics of sepsis patients at baseline and a comparison of the clinical characteristics between sepsis patients with IV vitamin C and sepsis patients without IV vitamin C. Most patients were male (64%), with a mean age of 65 years. A total of 32% of patients had hypertension, and 32% had diabetes. In the characteristics of treatment, 100% had a prescription for antibiotics, and 88% used glucocorticoids. Patients treated with IV vitamin C compared to those treated without IV vitamin C had a lower frequency of using glucocorticoids (75% vs. 100%, *p* = 0.039), higher SOFA scores (11.88 ± 2.75 vs. 10.33 ± 1.14, *p* = 0.05), and higher levels of platelets (256.88 ± 95.84 vs. 198.22 ± 69.94, *p* = 0.048); no statistically significant differences were found for the other parameters.

Table 2 depicts the comparison of changes in the clinical characteristics between sepsis patients treated with IV vitamin C and those treated without IV vitamin C at follow-up. We found that patients treated with IV vitamin C had a sharper decease in their SOFA scores (62% vs. 27%, *p* = 0.04) and higher platelet values (298.0 ± 115.09 vs. 217.56 ± 116.1, *p* = 0.05). On the other hand, we observed that patients treated without IV vitamin C had a higher 30-day mortality rate compared to patients treated with IV vitamin C as an adjuvant for the treatment of sepsis (55.6% vs. 18.8%, *p* = 0.03). We did not observe differences between the two groups of treatment in the number of days spent in the ICU, the total time from stay at hospital, the time spent using mechanical ventilation or the time that mechanical ventilation was stopped at 5 days, the number of days spent using vasoactive amines or the time that vasoactive amine use was reduced at 5 days, and the SOFA score at 72 h.

Table 3 shows the risk factors for mortality in patients with sepsis who were included in this study. We observed that treatment with vitamin C was protective for mortality in sepsis (RR: 0.54, 95% CI: 0.31–0.96, *p* = 0.03). On the other hand, the necessary use of vasoactive amines was a risk factor for mortality in these patients (RR:4.76, 95% CI:1.59–14.22, *p* = 0.001). We did not observe other factors associated with mortality in these patients.

## 4. Discussion

Our main objective was to identify whether treatment with IV vitamin C decreases the risk of mortality in patients with sepsis. We observed that the use of IV vitamin C as an adjuvant in the treatment of sepsis decreased the risk of mortality by 46% compared to those who were not treated with IV vitamin C as an adjuvant.

We observed a mortality of 38% in our patients at 30 days, which is similar to data reported in the literature. The frequency of mortality in sepsis varies from 3 to 47% and depends on a large number of factors; however, the average mortality rate found in studies on sepsis is approximately 24% at 30 days after diagnosis [12].

Only a few studies have assessed the effect of adding IV vitamin C to sepsis treatment. In a clinical trial, Lamontagne et al. observed no differences in 28-day mortality between patients with sepsis treated with IV vitamin C supplements plus thiamine and glucocorticoids and a placebo group [7]. Similarly, Sevransky et al. [13] and Mitchell et al. [14] did not observe differences in mortality in a group receiving IV vitamin C plus thiamine and hydrocortisone versus a placebo group. For his part, Wacker [15] conducted a study using IV vitamin C as a monotherapy; however, no differences in mortality were found in those patients who were treated with IV vitamin C compared to those who were treated without. These differences may be because we did not include patients with sepsis of a surgical origin or account for differences in the dosage of IV vitamin C; we used a standard dose of 2 g for all patients, administered with thiamine and glucocorticoids, as well as the base treatment. On the other hand, Mohamed et al. [16] carried out a clinical trial using triple therapy versus placebo, following their patients for 60 days, and found no differences in mortality between the groups. Alternatively, Marik et al. [17] observed reduced mortality in patients with severe sepsis and septic shock in a retrospective cohort study that compared treatment with IV vitamin C, thiamine, and hydrocortisone and a control group. Real-life studies are necessary due to the controversy surrounding current results observed in different studies. This is one of the first cohort studies that evaluates the independent use of IV vitamin C as an adjuvant in the management of patients with sepsis.

To date, various meta-analyses have been carried out evaluating the use of IV vitamin C as an adjuvant in the management of sepsis; Patel et al. [8], similar to our results, observed a decrease in the mortality rate with the use of IV vitamin C at high doses as monotherapy (RR: 0.64). We also identified a protective effect for 30-days mortality of using IV vitamin C (RR: 0.54). However, in Patel’s meta-analysis, when the results of studies using IV vitamin C in combined therapy with thiamine and glucocorticoids were analyzed, the authors did not find differences in decreases in the mortality [8]. Wen et al. [9], in their meta-analysis, identified a mild decrease in short-term mortality (RR: 0.82) and overall mortality (RR: 0.86); however, neither of these outcomes achieved statistical significance (*p* = 0.07 and *p* = 0.06, respectively). Sato et al. [10], in a meta-analysis, did not identify an association between the use of IV vitamin C and the decrease in the short-term mortality in patients with sepsis (RR: 0.88, *p* = 0.18). Cai et al. [11] differs from our results, and in their meta-analysis, the authors identify that the use of IV vitamin C in patients with sepsis does not reduce the in-hospital mortality rate (*p* = 0.27) or the ICU mortality rate (*p* = 0.07). The results of these meta-analyses may vary due to the characteristics of the patients and the clinical status at the time of inclusion, since they have variations in the SOFA score, age, and the IV vitamin C doses used.

We also observed that the use of IV vitamin C as an adjuvant was a protective factor for mortality compared to patients who were not treated with vitamin C (RR: 0.54). This result was similar to that observed by Marik et al. [17], who found that the use of IV vitamin C is a protective factor for mortality (RR: 0.13) in patients treated with IV vitamin C compared to those treated without. The differences in outcomes may be because Marik et al. [17] used a combined treatment with thiamine and hydrocortisone in conjunction with IV vitamin C. However, this differs from the results of Lamontagne et al. [7] and Lyu et al. [18], who both found a higher risk of mortality for patients who used IV vitamin C compared to a placebo group (RR: 1.17 and HR: 1.08), but these results were not statistically significant.

We observed that patients who required the use of vasoactive amines have a higher mortality risk (RR: 4.76) than patients who do not require them. This is similar to the findings of Ren Y et al. [19], who conducted a retrospective study in patients with sepsis and lung infection, identifying that the use of vasopressors was a risk factor for mortality (OR: 1.85); however, they did not reach statistical significance. The use of the vasoactive amines in patients with sepsis indicates a more serious systemic condition, which may lead to death.

In our study, we observed that the group treated with IV vitamin C presented a significant decrease in SOFA scores compared to the group treated without IV vitamin C (62.5% vs. 27.8%). This was contrary to the findings of Hwang et al. [20], who, in their study carried out in Korea, did not find differences in SOFA scores between the treatment group and the placebo group. However, Sato et al. identified, in their meta-analysis, that the use of IV vitamin C was associated with a decrease in SOFA score (*p* <0.01), which agrees with our results [10]. They used a combined treatment of IV vitamin C and thiamine in patients with septic shock, whereas our study used only a monotherapy with IV vitamin C as an adjuvant in the treatment of patients with sepsis and septic shock.

Vitamin C has an antioxidant effect, reducing the formation of free radicals and endothelial damage, as well as the permeability of the vascular endothelium and cell apoptosis. Likewise, it contributes to the formation of vasopressor substances and the vascular responses to these substances. In addition, vitamin C participates in the regulation of the immune system by reducing the release of proinflammatory factors by macrophages. On the other hand, it has been observed that using high doses of vitamins can also cause bacteriostatic activity, contributing to the management of infections [5].

We evaluated the effects of adding IV vitamin C to the conventional treatment of patients with sepsis in a prospective cohort on decreasing the mortality rates and other outcomes. This study involved a deferens variable analysis to determine the risk of mortality in these patients.

However, this study also has several limitations: patients with sepsis were evaluated from a single center, a limited sample size was used to detect other potential factors associated with outcomes, and there was a wide heterogeneity in therapeutic management. Nevertheless, this is a real-world study representing patients who are commonly treated in an intensive care unit; therefore, it has a good generalizability.

## 5. Conclusions

The incidence of mortality in our patients with sepsis was 38%. The addition of IV vitamin C to the conventional treatment of sepsis decreased the risk of mortality in these patients, who had SOFA scores of ≥9, by 46% compared to patients not treated with IV vitamin C as an adjuvant for sepsis. More follow-up studies with multi-center studies are necessary to clarify the benefits of vitamin C as an adjuvant in the treatment of sepsis from other causes.

## Figures and Tables

**Table 1:** Comparison of clinical characteristics between sepsis patients treated with vitamin C and those treated without vitamin C.

Variable	Total of Patients	Conventional Treatment n = 18	Vitamin C + Conventional Treatment n = 16	*p*
Age, years	65.06 ± 14.16	61.94 ± 14.84	68.56 ± 12.93	0.18
Male sex, n (%)	22 (64.7)	10 (55.6)	12 (75)	0.24
Diabetes, n (%)	9 (26.5)	4 (22.2)	5 (31.3)	0.70
Hypertension, n (%)	11 (32.4)	7 (38.9)	4 (25)	0.39
Chronic Kidney disease, n (%)	3 (8.8)	3 (16.7)	0 (0)	0.23
Hypothyroidism, n (%)	2 (5.9)	2 (11.1)	0 (0)	0.49
Antibiotic use, n (%)	34 (100)			
Glucocorticoids use, n (%)	30 (88.2)	18 (100)	12 (75)	**0.04**
Vitamin C use, n (%)	16 (47)			
Mechanical ventilation use, n (%)	20 (58.8)	10 (55.6)	10 (62.5)	0.68
Vasoactive amines use, n (%)	14 (41.2)	9 (50)	5 (31.3)	0.27
SOFA * score baseline	11.06 ± 2.17	10.33 ± 1.14	11.88 ± 2.75	**0.05**
MAP ** score baseline, units	72.85 ± 14.45	71.50 ± 9.92	74.38 ± 18.52	0.57
Platelet baseline	225.82 ± 87.05	198.22 ± 69.94	256.88 ± 95.84	**0.05**
Creatinine baseline	2.19 ± 3.14	2.80 ± 4.16	1.51 ± 1.10	0.22

Quantitative variables are expressed as means ± SD, and qualitative variables are expressed in frequencies (%). Comparison of qualitative variables was performed using Chi-square test. Comparisons of quantitative variables were carried out with Student *t*-test. Statistical significance was considered at *p* ≤ 0.05 level. * SOFA: sequential organ failure assessment; ** MAP: mean arterial pressure.

**Table 2:** Comparison of clinical characteristics between sepsis patients with vitamin C + conventional treatment and sepsis without vitamin C at follow-up.

Variable	Conventional Treatment n = 18	Vitamin C + Conventional Treatment n = 16	*p*
Total antibiotics use, units	2.17 ± 1.29	1.75 ± 0.77	0.26
Time of antibiotics use, days	14.78 ± 9.59	10.00 ± 5.03	0.08
ICU length of stay, days	11.44 ± 7.39	9.81 ± 5.32	0.46
Hospital length of stay, days	15.33 ± 9.86	11.75 ± 5.37	0.19
Time of mechanical ventilation, days	3.89 ± 5.68	4.50 ± 5.57	0.75
Mechanical ventilation stopped at 5 days	5 (55.6)	4 (44.4)	1.0
Use time of amines, days	2.89 ± 4.01	1.94 ± 3.38	0.46
Vasoactive amine reduction at 5 days	5 (71.4)	2 (28.6)	1.0
SOFA * score at 72 h, mid (range)	11 (6–17)	11 (7–18)	0.99
Decrease in SOFA (after 72 h)	5 (27.8)	10 (62.5)	**0.04**
MAP ** 72 h, units	78.89 ± 15.66	82.63 ± 13.14	0.46
Platelets 72 h	217.56 ± 116.10	298.00 ± 115.09	**0.05**
Creatinine 72 h	1.36 ± 1.60	1.11 ± 0.66	0.57
30-day mortality rate, n (%)	10 (55.6)	3 (18.8)	**0.03**

Quantitative variables are expressed as means ± SD, and qualitative variables are expressed in frequencies (%). Comparison of qualitative variables was performed using Chi-square test. Comparisons of quantitative variables were made with Student *t*-test. Statistical significance was considered at *p* ≤ 0.05 level. * SOFA: sequential organ failure assessment; ** MAP: mean arterial pressure.

**Table 3:** Factors associated with mortality in patients with sepsis.

Variable	RR	95% CI	*p*
Vitamin C use	0.54	0.31–0.96	**0.03**
Vassopresor amines use	4.76	1.59–14.22	**0.001**
Female	0.55	0.186–1.622	0.29
Hypertension	0.93	0.365–2.363	1.0
Diabetes Mellitus	1.74	0.766–3.931	0.25

Relative Risk, 95% CI, statistical significance *p* ≤ 0.05.

## Data Availability

Data to support the findings of this study are available upon reasonable request.
